# Distribution of Non-Persistent Endocrine Disruptors in Two Different Regions of the Human Brain

**DOI:** 10.3390/ijerph14091059

**Published:** 2017-09-13

**Authors:** Thomas P. van der Meer, Francisco Artacho-Cordón, Dick F. Swaab, Dicky Struik, Konstantinos C. Makris, Bruce H. R. Wolffenbuttel, Hanne Frederiksen, Jana V. van Vliet-Ostaptchouk

**Affiliations:** 1 Department of Endocrinology, University of Groningen, University Medical Center Groningen, 9713 GZ Groningen, The Netherlands; tomvandermeer20@gmail.com (T.P.v.d.M.); bwo@umcg.nl (B.H.R.W.); 2 Univ. Granada, Radiology and Physical Medicine Dept./ibs.GRANADA, 18016 Granada, Spain; fartacho@ugr.es; 3 Netherlands Institute for Neuroscience, an Institute of the Royal Netherlands Academy of Arts and Sciences, 1105 BA Amsterdam, The Netherlands; d.f.swaab@nin.knaw.nl; 4 Section of Molecular Metabolism and Nutrition, Department of Pediatrics, University of Groningen, University Medical Center Groningen, 9713 GZ Groningen, The Netherlands; d.struik@umcg.nl; 5 Cyprus International Institute for Environmental and Public Health, Cyprus University of Technology, Limassol 3041, Cyprus; konstantinos.makris@cut.ac.cy; 6 Department of Growth and Reproduction, Rigshospitalet, Copenhagen University Hospital, Blegdamsvej 9, DK-2100 Copenhagen, Denmark; Hanne.Frederiksen@regionh.dk

**Keywords:** bisphenol-A, methylparaben, phenols, parabens, brain, hypothalamus, obesity

## Abstract

Non-persistent endocrine disrupting chemicals (npEDCs) can affect multiple organs and systems in the body. Whether npEDCs can accumulate in the human brain is largely unknown. The major aim of this pilot study was to examine the presence of environmental phenols and parabens in two distinct brain regions: the hypothalamus and white-matter tissue. In addition, a potential association between these npEDCs concentrations and obesity was investigated. Post-mortem brain material was obtained from 24 individuals, made up of 12 obese and 12 normal-weight subjects (defined as body mass index (BMI) > 30 and BMI < 25 kg/m^2^, respectively). Nine phenols and seven parabens were measured by isotope dilution TurboFlow-LC-MS/MS. In the hypothalamus, seven suspect npEDCs (bisphenol A, triclosan, triclocarban and methyl-, ethyl-, n-propyl-, and benzyl paraben) were detected, while five npEDCs (bisphenol A, benzophenone-3, triclocarban, methyl-, and n-propyl paraben) were found in the white-matter brain tissue. We observed higher levels of methylparaben (MeP) in the hypothalamic tissue of obese subjects as compared to controls (*p =* 0.008). Our findings indicate that some suspected npEDCs are able to cross the blood–brain barrier. Whether the presence of npEDCs can adversely affect brain function and to which extent the detected concentrations are physiologically relevant needs to be further investigated.

## 1. Introduction

In recent decades, industrial progress has introduced exposure to a huge number of new synthetic chemicals—e.g., environmental phenols and parabens such as bisphenol A (BPA), triclosan (TCS), and benzophenone-3 (BP-3)—that can be found in a wide variety of commercial products including plastics, packaged food and drinks, personal care products, and pharmaceuticals [1,2]. Humans are exposed to these chemicals via ingestion, inhalation, dermal contact, and perinatal transmission (i.e., placenta, breast milk) [3,4,5], and the exposure is ubiquitous in western populations [5,6,7,8,9]. Some environmental phenols and parabens are labeled as non-persistent endocrine disrupting chemicals (npEDCs) because of their known or suspected adverse effects on endocrine and metabolic regulation [10,11] and quick metabolism and excretion from the body [2]. However, traces of these npEDCs have recently been detected in human adipose tissue, liver, and brain [12,13,14,15].

Since the early 1980s, the prevalence of obesity has more than doubled worldwide, to 600 million obese adults in 2014 [16]. This increase is reaching epidemic proportions, and can no longer be solely explained by genetic predisposition, increased caloric intake, and lack of physical activity [17]. Accumulating evidence from epidemiological studies suggests involvement of npEDCs in the increased prevalence of obesity [18,19,20,21,22]. Also, the obesogenic properties of npEDCs are supported by functional and in vitro studies [17].

While a lot of research concerning npEDCs and obesity has been done over the years, very little is known about its presence in and potential effects on the human brain. Energy balance consists of complex homeostatic mechanisms involving both peripheral organs and the brain [23], with the hypothalamus playing a central role in the regulation of energy expenditure, metabolism, and nutrient partitioning [24]. Because the hypothalamus receives information through circulating metabolites and hormones, and is therefore susceptible to a wide variety of hormones [24], it is plausible that some EDCs have the potential to infiltrate there and interfere with the physiological processes. Recently, exposure to low doses of BPA in zebrafish has been shown to increase hypothalamic neurogenesis [25]. In rodents, perinatal exposure to BPA has been shown to disrupt the signaling of multiple regulatory hormones, including leptin and insulin [26], very likely through developmental programming of the hypothalamic melanocortin system, permanently remodeling the neurobiology of metabolic homeostasis [27].

As it is only partly protected by the blood–brain barrier (BBB) [28], the hypothalamic exposure to npEDCs is expected to differ from BBB-protected brain regions, such as the white-matter tissue [28]. Although there are some indications of npEDCs presence in human brain tissue [13], information on the distribution of npEDCs in the human brain is not available yet. The major aim of this pilot study was to examine the presence of environmental phenols and parabens in two distinct brain regions, the hypothalamus and white-matter tissue. In addition, we also investigated a potential association between these npEDCs concentrations and obesity.

## 2. Methods

We used frozen hypothalamic and white-matter brain tissue material obtained from The Netherlands Brain Bank (NBB), Netherlands Institute for Neuroscience, Amsterdam (open access: www.brainbank.nl). All material was collected from donors whose written informed consent for a brain autopsy and the use of their brain material and clinical information regarding research purposes had been obtained by the NBB [29]. Hypothalamus samples came from 24 individuals (Table S1), 12 of whom were normal-weight controls and 12 were obese individuals (cases) (body mass index (BMI) < 25 kg/m^2^ and BMI > 30 kg/m^2^, respectively) that were matched for sex, age, clinical diagnosis, and Braak stage of Alzheimer’s progression [30]. Additional white-matter lipid-enriched brain tissue was collected from five of the above-mentioned matched case-control pairs. All procedures were performed in accordance with national and institutional guidelines and with the ethical guidelines of the Declaration of Helsinki.

Seven parabens (methyl paraben (MeP), ethyl paraben (EtP), iso-propyl paraben (i-PrP), n-propyl paraben (n-PrP), iso-butyl paraben (i-BuP), n-butyl paraben (n-BuP) and benzyl paraben (BzP)) and nine phenols (BPA, TCS, triclocarban (TCC), BP-3, 2,4-dichlorophenol (2.4-DCP), 2,5-dichlorophenol (2.5-DCP), 2,4,5-trichlorophenol (2.4.5-TCP), 2-phenylphenol (2-PP) and 4-phenylphenol (4-PP)) were analyzed by means of TurboFlow-LC-MS/MS at Department of Growth and Reproduction, Rigshospitalet, Copenhagen with a recently developed method for adipose tissue [12]. Sample weights varied between 44.7 mg and 118.9 mg, and all chemicals, solutions, and laboratory wares were checked for contamination before use. All samples were processed as described previously [12]. In short, samples were analyzed in batches including standards for calibration curves, unknown samples, two blanks, and two control samples (a pool of human fatty tissues) spiked at low and high levels.

Descriptive statistics (mean, Standard Deviation (SD), median, percentiles, minimum and maximum) of the npEDCs were calculated for a total population and stratified by status. The compounds detected in >50% of the samples (BPA and MeP) were included in further analysis, in which concentrations below the limit of detection (LOD) were replaced by LOD/√2. The correlation between BPA and MeP levels and age, body weight, BMI, and other parameters was examined using Spearman’s rho test. Differences between groups were statistically evaluated by the unpaired *t*-test with Welch’s correction or by the Wilcoxon matched paired signed-rank test. Tests were two-tailed. The level of a nominal significance was set at *p <* 0.05. Optimal sample sizes were calculated using NCSS PASS software version 11.0 [31,32], assuming that the observed means and standard deviations were representative of the target population. We furthermore assumed a normal distribution of BPA levels, and normal distribution of paired differences in BPA levels between hypothalamus and white-matter tissue. Statistical analysis was conducted with SPSS (version 22 for Windows, SPSS Corporation, Chicago, IL, USA). Graphs were computed with GraphPad Prism software for Windows, Version 5 (GraphPad Software, Inc., La Jolla, CA, USA).

## 3. Results

**Figure 1:**
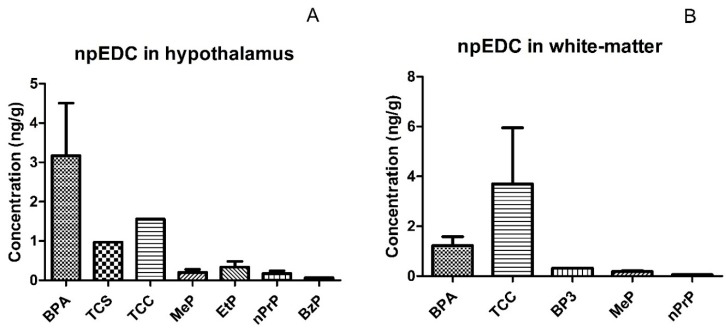
Measured concentrations (mean ± SD) of npEDC in hypothalamus, *n* = 24 (**A**) and white-matter brain tissue, *n* = 12 (**B**). Abbreviations: BPA: bisphenol A; BP-3: benzophenone-3; TCC: triclocarban; TCS: triclosan; MeP: methylparaben; EtP: ethylparaben; npEDCs: non-persistent endocrine disrupting chemicals; n-PrP: n-propylparaben; BzP: benzylparaben; SD: standard deviation.

In this study, we included hypothalamic and white-matter brain tissues post mortem obtained from 12 obese and 12 normal-weight control subjects. The study population consisted mainly of women (67%) and the mean age was 74 years old. There were no differences in sex, age, and brain weight between cases and controls (Table S2). A descriptive analysis of the npEDCs measured is presented, for both hypothalamus and white-matter, in Table 1. Out of the nine phenols and seven parabens analyzed, three phenols (BPA, TCS, and TCC) and four parabens (MeP, EtP, n-PrP, and BzP) were detected in the hypothalamus (Figure 1A), while in white-matter brain tissue we found three phenols (BPA, BP-3, and TCC) and two parabens (MeP and n-PrP) (Figure 1B). Of the 24 hypothalamic samples, BPA was detectable above LOD in 23 samples, MeP in 15 samples, EtP in 3 samples, and n-PrP in 5 samples. BPA, TCC, and MeP were detectable in respectively 9, 2, and 3 of the 12 white-matter samples. Furthermore, TCS, TCC, and BzP were detected in single hypothalamus samples, while BP-3 and n-PrP were detectable in single white-matter samples (Table 1). The ranges of some npEDCs in brain tissues were wide, with the largest spread in the concentration found for BPA in both hypothalamus and white-matter (between 0.32 and 26.62 ng/g and between 0.30 and 3.32 ng/g, respectively), for MeP in hypothalamus (ranging between 0.06 and 1.16 ng/g) and for TCC in white-matter tissue (ranging between 1.45 and 5.95 ng/g).

**Figure 2:**
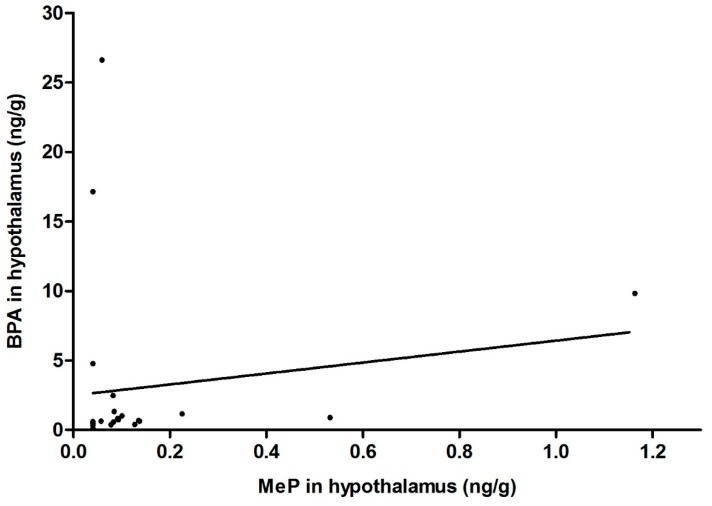
Correlation between BPA and MeP concentrations in the hypothalamus (Spearman correlation coefficient *r =* 0.37, *p =* 0.078).

We observed a trend towards a positive correlation between BPA and MeP levels in the hypothalamus (correlation coefficient *r* = 0.37, *p* = 0.078) (Figure 2), with BPA concentrations being significantly higher than those of MeP (median: 0.68 vs. 0.09 ng/g, *p* = 0.038, respectively).

**Figure 3:**
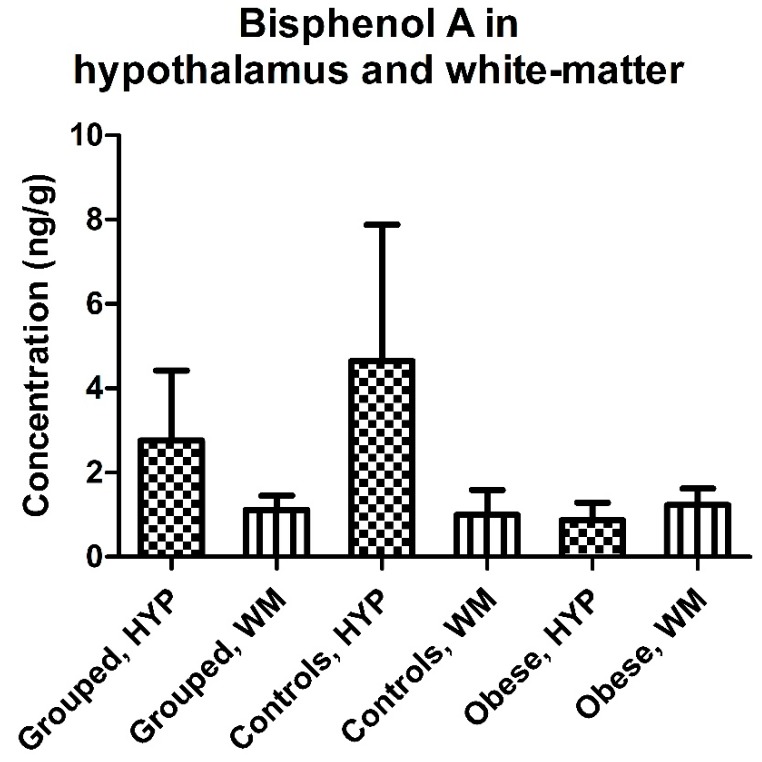
Concentrations (mean ± SD) of bisphenol A (BPA) in hypothalamus and white-matter brain tissue in all paired samples combined, controls, and obese cases.

**Figure 4:**
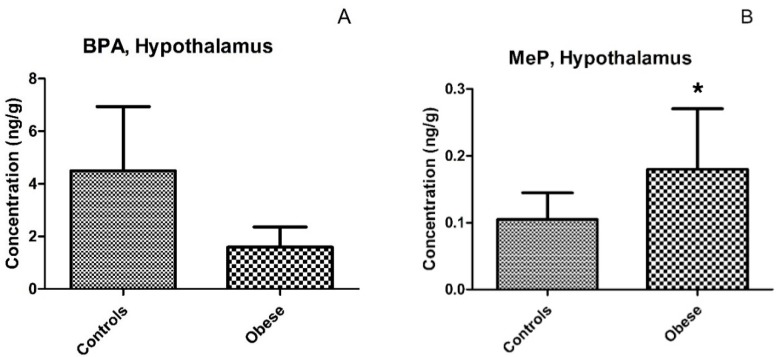
Concentrations (mean ± SD) of bisphenol A (BPA) (**A**) and methyl paraben (MeP) (**B**) in paired hypothalamus tissues from controls (*n* = 12) and obese individuals (*n* = 12). *- *p <* 0.05.

No differences were observed between hypothalamic and white-matter brain regions in terms of BPA or MeP levels (Table 2). A similar trend was observed in both controls and obese individuals with slightly but not significantly higher BPA levels in white-matter tissue than was found in the hypothalamus in the obese group, while an opposite pattern was found in controls (white-matter vs. hypothalamus; median (mean ± SD): 1.01 (1.23 ± 0.89) vs. 0.54 (0.87 ± 0.92) ng/g, *p* = 0.62, in obese, and 0.38 (1.00 ± 1.32) vs. 0.59 (4.65 ± 7.23) ng/g, *p* = 0.63, in controls) (Figure 3). The BPA concentrations were similar for obese and normal-weight individuals in both hypothalamus and white-matter tissues (obese vs. controls; median (mean ± SD): in white-matter 1.01 (1.23 + 0.89) vs. 0.38 (1.00 + 1.32) ng/g, *p =* 0.81, and in the hypothalamus 0.71 (1.59 ± 2.66) vs. 0.63 (4.49 ± 8.46) ng/g, *p* = 0.79) (Table 2, Figure 4A). We detected a significantly higher MeP concentration in the hypothalamus of obese individuals as compared with controls (median (mean ± SD): 0.08 (0.18 ± 0.31) vs. 0.05 (0.10 ± 0.14), *p =* 0.008) (Table 2, Figure 4B).

Finally, we found no correlation between BPA or MeP levels and age, body weight, BMI, and brain weight, in both hypothalamic and white-matter tissue (data not shown).

## 4. Discussion

The detection of seven common environmental chemicals all suspected to be npEDCs—including BPA, TCS, TCC, MeP, EtP, nPrP and BzP—in the hypothalamus indicate the ability of these chemicals to infiltrate the hypothalamus with the potential to cause adverse health effects. In the white-matter brain tissue, five npEDCs (BPA, TCC, BP3, MeP, and nPrP) were detected, suggesting that some of the phenols and parabens might be able to cross the BBB barrier. Also, such a difference between the examined brain regions in terms of npEDC exposure may be explained by the BBB’s protection of white-matter, which is lacking for parts of the hypothalamus, as well as by the relatively high vascularity of the hypothalamus, due to its central role in the receiving of multiple hormonal signals [24].

Two npEDCs have previously been detected in human brain tissue [13]. Geens et al. reported BPA concentrations in brain tissue with a median of 0.57 ng/g [13], showing similar levels as in the present pilot study (0.68 ng/g in hypothalamus and 0.82 ng/g in white-matter). TCS was also measured in 1 (0.23 ng/g) out of 11 brain samples [13], while we detected TCS in one single hypothalamic sample (0.97 ng/g). To our knowledge, no other study has reported npEDC distribution in either human hypothalamus or white-matter brain tissues. In brain tissue samples, we observed a wide concentration range for BPA, MeP, and TCC, suggesting inter-individual differences in the exposure and/or its metabolism and excretion. Previous studies have also reported a high variability in concentrations for BPA in adipose tissue (ranges: 1.80–12.01 ng/g [14], 1.12–12.28 ng/g [13], and <the limit of quantification (LOQ)–20.9 ng/g [15]) and for MeP in adipose tissue (range: <LOQ–22.3 ng/g [15]), but not for BPA in brain (range: <LOD–2.36 ng/g [13]). The variation in individual npEDC exposure may be the result of a number of factors, including sex, age, disease status, and lifestyle. In the current study, the limited number of available brain samples and the modest collection of data did not yield evidence for such correlations.

We observed brain region-specific presence of some npEDCs that were detected only in the hypothalamus (TCS, EtP, and BzP) or white-matter tissue (BP-3). This suggests that the hypothalamus and the white-matter are susceptible to different npEDCs, and that the various npEDCs differ in terms of their ability to cross the BBB and/or a potential to accumulate in lipophilic brain tissue. In our study sample, we found no evidence for a difference in BPA concentration between hypothalamic and white-matter regions, neither when the data was stratified by obesity status. Determining whether or not there is a preferential accumulation of npEDCs that are able to cross the BBB in specific regions of the brain requires further investigation.

Since the hypothalamus is a major regulator of body weight [24,28], and some EDCs have been previously associated with obesity [11,17,18,21], we hypothesized that obese individuals might have higher levels of some EDCs compared to normal-weight individuals. We found significant association between higher levels of MeP measured in hypothalamus in obese individuals compared to the levels measured in normal-weight individuals. Although recent animal and in vitro studies have reported the obesogenic properties of MeP [33,34], from the present study design it is not possible to determine to which extent the detected relationship contributes to the development of obesity. For BPA, no significant differences between obese and normal-weight subjects were observed in terms of the chemical concentrations in both hypothalamus and white-matter tissue (*p* > 0.7). This could be due to the relatively small sample size, caused by the limited availability of post mortem brain material from obese individuals and well-matched controls as well as due to the above-mentioned high inter-individual variability in BPA levels. Assuming that the observed distribution in the hypothalamic BPA concentration between obese individuals and controls is representative of a target population, a sample size of 32 cases and 32 controls is required to find a significant difference of the same magnitude (i.e., such a sample size will provide 81% power to detect an observed difference with a significance level of 0.05 using a two-sided two-sample *t*-test).

Note that many individuals included in the present study were elderly and/or suffered from neurological disorders. Since age and neurodegenerative disorders can alter the BBB properties and permeability [35], we cannot exclude a potential confounding effect of these factors on the npEDC levels detected in the brain tissues. Follow-up studies in wider populations are warranted to clarify these questions and to assess the clinical relevance of our findings.

Finally, the detection of npEDCs in the hypothalamus raises important questions about their potential adverse effects on the metabolism. Previously, a strong association between the serum level of BPA and circulating adiponectin, leptin, and the gut-hormone ghrelin has been reported in humans, suggesting BPA interference with hormonal regulation of hunger and satiety [36]. How the presence of BPA and other npEDCs in the major center of metabolic regulation [24] is altering the physiological processes remains to be determined.

## 5. Conclusions

This study shows—for the first time—the distribution of three environmental phenols and four parabens, assumed to be non-persistent, in the human hypothalamus, indicating their ability to infiltrate, and their potential to accumulate in the brain region responsible for the regulation of metabolism. A smaller number of these chemicals were also detected in white-matter tissues, indicating that the BBB hinders access of chemicals to white-matter lipid fraction. Our results also suggest a possible relationship between MeP levels in the hypothalamus and obesity. Further research is needed to determine to which degree npEDCs might disrupt the normal physiological processes and functioning of the brain.

## Figures and Tables

**Table 1:** Phenols and parabens (ng/g) in hypothalamus and white-matter brain tissues.

			Hypothalamus (*n* = 24)								White-Matter Brain (*n* = 10)							
Compound		LOD (ng/g)	N (%) > LOD	Mean	SD	Median	Min	P25	P75	Max	N (%) > LOD	Mean	SD	Median	Min	P25	P75	Max
Phenols	BPA	0.14	23 (96)	3.17	6.42	0.68	0.32	0.53	1.23	26.62	9 (90)	1.23	1.07	0.82	0.30	0.38	1.90	3.32
	TCS	0.73	1 (4)	0.97	-	0.97	0.97	-	-	0.97	0	<LOD	-	<LOD	<LOD	<LOD	<LOD	<LOD
	TCC	0.92	1 (4)	1.56	-	1.56	1.56	-	-	1.56	2 (20)	3.70	3.18	3.70	1.45	1.45	5.95	5.95
	BP3	0.18	0	<LOD	-	<LOD	<LOD	<LOD	<LOD	<LOD	1 (10)	0.32	-	0.32	0.32	-	-	0.32
	2.4-DCP	0.10	0	<LOD	-	<LOD	<LOD	<LOD	<LOD	<LOD	0	<LOD	-	<LOD	<LOD	<LOD	<LOD	<LOD
	2.5-DCP	1.83	0	<LOD	-	<LOD	<LOD	<LOD	<LOD	<LOD	0	<LOD	-	<LOD	<LOD	<LOD	<LOD	<LOD
	2.4.5-TCP	0.49	0	<LOD	-	<LOD	<LOD	<LOD	<LOD	<LOD	0	<LOD	-	<LOD	<LOD	<LOD	<LOD	<LOD
	2-PP	0.10	0	<LOD	-	<LOD	<LOD	<LOD	<LOD	<LOD	0	<LOD	-	<LOD	<LOD	<LOD	<LOD	<LOD
	4-PP	1.31	0	<LOD	-	<LOD	<LOD	<LOD	<LOD	<LOD	0	<LOD	-	<LOD	<LOD	<LOD	<LOD	<LOD
Parabens	MeP	0.06	15 (63)	0.20	0.29	0.09	0.06	0.08	0.14	1.16	3 (30)	0.18	0.06	0.15	0.14	0.14	0.20	0.26
	EtP	0.06	3 (13)	0.34	0.26	0.36	0.07	0.21	0.47	0.58	0	<LOD	-	<LOD	<LOD	<LOD	<LOD	<LOD
	nPrP	0.05	5 (21)	0.17	0.15	0.12	0.05	0.08	0.20	0.41	1 (10)	0.06	-	0.06	0.06	-	-	0.06
	BzP	0.05	1 (4)	0.06	-	0.06	0.06	-	-	0.06	0	<LOD	-	<LOD	<LOD	<LOD	<LOD	<LOD
	i-PrP	0.05	0	<LOD	-	<LOD	<LOD	<LOD	<LOD	<LOD	0	<LOD	-	<LOD	<LOD	<LOD	<LOD	<LOD
	i-BuP	0.06	0	<LOD	-	<LOD	<LOD	<LOD	<LOD	<LOD	0	<LOD	-	<LOD	<LOD	<LOD	<LOD	<LOD
	n-BuP	0.08	0	<LOD	-	<LOD	<LOD	<LOD	<LOD	<LOD	0	<LOD	-	<LOD	<LOD	<LOD	<LOD	<LOD

BPA: bisphenol A; BP-3: benzophenone-3; TCC: triclocarban; TCS: triclosan; MeP: methylparaben; EtP: ethylparaben; n-PrP: n-propylparaben; BzP: benzylparaben; LOD: limit of detection; SD: standard deviation.

**Table 2:** Descriptive analysis of detected npEDC (ng/g) in hypothalamus and white-matter brain tissue by obesity status.

			Hypothalamus (*n* = 12 Controls, 12 Obese)						White-Matter Brain (*n* = 5 Controls, 5 Obese)					
Compound		Status	N (%) > LOD	Mean	Median	P25	P75	Max	N (%) > LOD	Mean	Median	P25	P75	Max
Phenols	BPA	controls	12 (100)	4.49	0.63	0.48	2.89	26.62	4 (80)	1.22	0.60	0.38	2.07	3.32
		obese	11 (92)	1.73	0.73	0.56	1.23	9.82	5 (100)	1.23	1.01	0.53	1.90	2.39
	TCS	controls	0	<LOD	<LOD	<LOD	<LOD	<LOD	0	<LOD	<LOD	<LOD	<LOD	<LOD
		obese	1 (8)	0.97	0.97	-	-	0.97	0	<LOD	<LOD	<LOD	<LOD	<LOD
	TCC	controls	0	<LOD	<LOD	<LOD	<LOD	<LOD	1 (20)	5.95	5.95	-	-	5.95
		obese	1 (8)	1.56	1.56	-	-	1.56	1 (20)	1.45	1.45	-	-	1.45
	BP3	controls	0	<LOD	<LOD	<LOD	<LOD	<LOD	1 (20)	0.32	0.32	-	-	0.32
		obese	0	<LOD	<LOD	<LOD	<LOD	<LOD	<LOD	<LOD	<LOD	<LOD	<LOD	<LOD
Parabens	MeP	controls	6 (50)	0.17	0.11	0.06	0.14	0.53	1 (20)	0.26	0.26	-	-	0.26
		obese	9 (75)	0.23	0.09	0.08	0.14	1.16	2 (40)	0.15	0.15	0.14	0.15	0.15
	EtP	controls	2 (17)	0.32	0.32	0.07	0.58	0.58	0	<LOD	<LOD	<LOD	<LOD	<LOD
		obese	1 (8)	0.36	0.36	-	-	0.36	0	<LOD	<LOD	<LOD	<LOD	<LOD
	nPrP	controls	3 (25)	0.13	0.12	0.10	0.16	0.20	0	<LOD	<LOD	<LOD	<LOD	<LOD
		obese	2 (17)	0.23	0.23	0.05	0.41	0.41	1 (20)	0.06	0.06	-	-	0.06
	BzP	controls	0	<LOD	<LOD	<LOD	<LOD	<LOD	0	<LOD	<LOD	<LOD	<LOD	<LOD
		obese	1 (8)	0.06	0.06	-	-	0.06	0	<LOD	<LOD	<LOD	<LOD	<LOD

BPA: bisphenol A; BP-3: benzophenone-3; TCC: triclocarban; TCS: triclosan; MeP: methylparaben; EtP: ethylparaben; n-PrP: n-propylparaben; BzP: benzylparaben; LOD: limit of detection; SD: standard deviation.

## Supplementary Materials

The following are available online at www.mdpi.com/1660-4601/14/9/1059/s1, Table S1: Clinicopathological details of the subjects with available hypothalamic material (*n* = 24), Table S2: Basic characteristics of the study population.
