# Supplementary material for: Distribution of Non-Persistent Endocrine Disruptors in Two Different Regions of the Human Brain
Source: Int J Environ Res Public Health. 2017 Sep 13;14(9):1059. doi: 10.3390/ijerph14091059 (PMC5615596; doi:10.3390/ijerph14091059)
Supplement: Supplementary file 1 [file ijerph-14-01059-s001.pdf]

International Journal of
Environmental Research and Public Health

MDPI

Supplementary Materials

Distribution of Non-Persistent Endocrine Disruptors in Two Different Regions of the Human Brain

Thomas P. van der Meer, Francisco Artacho-Cordón, Dick F. Swaab, Dicky Struik, Konstantinos C. Makris, Bruce H. R. Wolffenbuttel, Hanne Frederiksen, and Jana V. van Vliet-Ostaptchouk

Table S1. Clinicopathological details of the subjects with available hypothalamic material (n = 24).

Number	White matter	Age (years)	Sex	BMI (kg/m^2)	PMD (hr:min)	Year of death	Braak stage	Clinical diagnosis and cause of death
Control group								
1	No	82	f	22.9	05:25	2009	5	Alzheimer's disease
2	No	83	f	24.0	04:05	2010	1	Ischemic changes
3	No	71	m	20.1	04:00	2010	6	Alzheimer's disease
4	No	82	f	20.3	03:55	2011	2	Parkinson's disease
5	No	84	f	24.6	06:33	2012	5	Alzheimer's disease
6	No	47	m	22.7	05:25	2013	-	Fronto-temporal dementia
7	Yes	75	f	24.7	04:50	2002	1	Non-Alzheimer dementia
8	Yes	47	f	21.3	04:25	2007	1	Multiple sclerosis
9	Yes	55	m	22.6	06:20	2008	-	Multiple sclerosis
10	Yes	79	m	20.6	05:00	2010	3	Lewy bodies variant
11	Yes	84	f	23.4	06:45	2012	2	Vascular dementia
12	Yes	86	f	24.0	05:15	2013	6	Alzheimer's disease
Obese group								
13	No	84	f	33.9	05:15	2000	5	Alzheimer's disease
14	No	49	m	31.2	04:55	2007	-	Fronto-temporal dementia tauopathy
15	No	81	f	36.2	05:55	2011	4	Parkinson's disease
16	No	84	f	31.2	05:36	2012	2	Non-demented control
17	No	81	f	31.1	04:00	1998	5	Alzheimer's disease
18	No	73	m	33.2	05:30	1999	4	Alzheimer's disease
19	Yes	84	f	36.6	05:10	2002	1	Dementia
20	Yes	88	f	31.6	03:15	2002	5	Alzheimer's disease
21	Yes	77	f	35.3	04:30	2009	0	Fronto-temporal dementia
22	Yes	84	m	31.6	07:20	2009	1	Non-demented control with Lewy bodies
23	Yes	56	m	30.4	09:35	2013	0	Multiple sclerosis
24	Yes	53	f	31.2	07:15	2013	-	Multiple sclerosis

BMI: body mass index, F: female; hr: hour; M: male; min: minutes; PMD: post mortem delay. * for Braak stage see the reference Braak, H.; Braak, E. Neuropathological stageing of alzheimer-related changes. *Acta Neuropathol.* **1991**, *82*, 239–259.

Table S2. Basic characteristics of the study population.

**Characteristics**	**Controls (n = 12)**	**Obese individuals (n = 12)**
Sex (men (N,%))	4 (33)	4 (33)
Age (years)	72.9 ± 14.7	74.5 ± 13.8
Weight (kg) *	64.2 ± 9.0	86.2 ± 7.9
BMI (kg/m^2) *	22.6 ± 1.7	32.8 ± 2.2
Brain weight (kg)	1175.2 ± 119.9	1131 ± 129.9

Data are presented as mean ± SD. $*p < 2.0\text{x}10^{-6}$
